# Floors and Walls of Operating-Theatres

**Published:** 1921-09-17

**Authors:** R. Langton Cole

**Affiliations:** 23 Throgmorton Street, E.C. 2


*To the Editor of* The Hospital.

### Floors and Walls of Operating-Theatres.

*To the Editor of* The Hospital.

Sir,—As the result of experience extending over a large number of years, and of recent conferences with those having the use of operating-theatres, I do not wholly agree with the statement made in your issue of September 3.

My opinion, for what it is worth, is that the floors of operating-theatres should be made of some jointless material, of which there are now many in the market. It is better to stop up a crack occasionally than to have the floor covered with small inequalities, as is the case with any form of terrazzo.

As regards the walls, in my opinion, plaster, painted with enamel, is the most satisfactory. I have used glazed opalite tiles, which give a beautiful surface, but the joints between, although small, turn *black*, and there must be some reason for this blackness.

The use of plaster also permits of the heating pipes being buried in the walls. Certain difficulties may be raised by this combination, but, on the whole, it appears to have very distinct advantages.—Yours faithfully,

R. Langton Cole.

23 Throgmorton Street, E.C. 2,

September 8, 1921.

